# Prevalence and social determinants of psychological distress among people who use drugs in Cambodia

**DOI:** 10.1186/s13033-020-00411-5

**Published:** 2020-11-04

**Authors:** Chan Hang Saing, Kiesha Prem, Ponha Uk, Navy Chann, Pheak Chhoun, Phalkun Mun, Sovannary Tuot, Siyan Yi

**Affiliations:** 1 Saw Swee Hock School of Public Health, National University of Singapore and National University Health System, 12 Science Drive 2, #10-01, Singapore, 117549 Singapore; 2 Department of Infectious Disease Epidemiology, Faculty of Epidemiology and Population Health, London School of Hygiene & Tropical Medicine, London, UK; 3 National Center for HIV/AIDS, Dermatology and STD, Phnom Penh, Cambodia; 4 KHANA Center for Population Health Research, Phnom Penh, Cambodia; 5 Center for Global Health Research, Touro University California, Vallejo, CA USA; 6 School of Public Health, National Institute of Public Health, Phnom Penh, Cambodia

**Keywords:** Substance use, Mental health, Adverse childhood experiences, Resource-limited setting, Asia

## Abstract

**Background:**

People who use drugs are at a disproportionately higher risk of mental disorders due to prolonged exposure to psychosocial challenges. However, studies on mental health among people who use drugs in resource-constrained countries are scarce. This study sheds light on the prevalence and correlates of psychological distress among people who use drugs in Cambodia.

**Methods:**

We conducted this cross-sectional study in the capital city and 11 provinces in 2017. The Respondent Driven Sampling method was adapted to recruit 1677 people who used drugs for face-to-face interviews using a structured questionnaire. Psychological distress was measured using the General Health Questionnaire (GHQ-12). A total score of GHQ-12 > 2 indicated high psychological distress. We performed a multiple logistic regression analysis to identify factors associated with psychological distress.

**Results:**

We included 1598 participants in the analyses, with a mean age of 28.6 years (SD = 7.8). Of the total, 42% had high psychological distress – 50% in women and 37% in men. The adjusted odds of having high psychological distress were significantly higher among participants who were 25–34 years old and 35 years and above, had been to a drug rehabilitation center, had been insulted by family members, and had been sexually harassed/abused by someone when they were growing up. The odds of having high psychological distress were significantly lower among participants who were male, lived in their own dwelling, reported injecting as the mode of the first drug use, and had someone taking care of them when they got sick.

**Conclusions:**

This study documents a high prevalence of psychological distress among people who use drugs in Cambodia. Intervention programs that attempt to address mental health problems among people who use drugs in resource-limited settings should be gender- and age-sensitive and target more marginalized subpopulations. Mental health services can be integrated into HIV and harm-reduction programs for people who use drugs.

## Background

In 2017, the global estimate of the number of people who use drugs, including people who inject drugs, aged 15–64 were 271 million [1], equivalent to about 5% of the world population of the same age. Of them, about 35 million (13%) suffered from drug-use disorders, which resulted in approximately 166,613 deaths and a loss of 27 million Disability-Adjusted Life Years (DALYs) [1, 2]. Therefore, drug use is recognized as one of the major global public health concerns. However, the availability of and access to treatment services among people with drug use disorders remain limited globally. Only one in seven people who use drugs receive the treatment each year [1].

Existing studies show that the comorbidity between drug-use disorders and mental health problems is common among people who use drugs. Moreover, people who use drugs without drug-use disorders are also at increased risk of mental health problems [3–10]. Substance use disorders have been found to occur in tandem with anxiety disorders including generalized anxiety disorder, panic disorder, and post-traumatic stress disorder [11–14]; mental disorders including depression and bipolar disorder [8, 11–13]; attention deficit hyperactivity disorder [12, 14]; and antisocial personality disorder [15].

The relationship between mental health and risky drug use, particularly intravenous drug use and unsafe sexual practices, has been well documented in previous studies. People who use drugs with poor mental health, such as severe depressive symptoms, are more likely to adopt unsafe injection practices such as sharing needles and syringes [16–18]. Previous studies have also reported that people who use drugs with mental health problems were more likely to have more sexual partners and engage in frequent condomless sexual intercourse than those without mental health problems [19, 20]. These risky behaviors are associated with a higher risk of acquiring human immunodeficiency virus (HIV) [21].

Mental health problems of people who use drugs are a significant public health concern as it co-occurs with drug use disorders and mediates other viral infections such as HIV and hepatitis C virus (HCV) [22–24]. Therefore, addressing risk factors associated with mental health problems among people who use drugs would reduce the disease burden of drug use disorders and HIV and HCV infections among people who use drugs. Previous studies have documented drug use behaviors and experiences such as duration [25], frequency [21], overdose [26–28], and drug rehabilitation [21, 25] as associated risk factors of psychological distress. Exposure to violence, such as stigma and discrimination and sexual assault, and lack of social support, such as family intimacy and adaptability, have also been predictors of psychological distress among people who use drugs [26, 29–34].

In Cambodia, the latest estimated number of people who use drugs aged 18 years and above in 2017 was notably large at around 22,374 people [16]. Previous studies show that psychological distress is common among Cambodian people who use drugs [17, 21, 25]. Also, access to psychological support, such as services provided in drop-in centers by community-based organizations, is limited [16]. In 2017, 90,672 people received mental health treatment within public health facilities, with approximately 5% reporting that their mental health condition was driven by substance use [35]. Our previous study showed that adverse childhood experiences (ACEs) were common and associated with psychological distress among people who use drugs in Phnom Penh [21]. Despite the common mental health problems and limited access to care among people who use drugs, studies on these issues remain scarce, preventing it from gaining policy and strategic attention.

To our knowledge, two studies on the mental health of people who use drugs have been conducted in Cambodia [21, 25]. Yi et al. included only people who inject drugs living in the capital city of Phnom Penh, while Heng et al. used data from the national survey conducted in 2012 and focused primarily on the relationship between mental health outcomes and history of drug rehabilitation. In this study, we used data from the most recent national integrated biological and behavioral survey in 2017 to examine the prevalence and correlates of psychological distress among people who use drugs in Cambodia.

## Methods

### Study design and setting

We conducted this cross-sectional study in Cambodia in 2017 in the capital city and 11 provinces. A feasibility assessment was conducted before selecting the 12 sites, which consisted of 21 operational districts with a high burden of HIV and a large population of people who use drugs. People who use drugs were defined as people who have used any illicit drugs, as stated in the Cambodian Law on Control of Drugs, in the past 12 months [36].

### Eligibility criteria

To be eligible for the survey, an individual must: (1) be at least 18 years old, (2) have a predetermined study coupon, (3) never participate in this survey earlier, (4) meet the definition of people who use drugs, and (5) be able and willing to provide written informed consent to participate in the survey. Details of the survey have been published elsewhere [37–39].

### Sampling procedure

We adapted the Respondent-Driven Sampling (RDS) method to recruit the study participants due to the hard-to-reach nature of people who use drugs. The sampling procedure included five steps. Initially, we sought support from local NGOs in the selected 21 operational districts to obtain four seeds with a good connection with people who use drugs in each operational district. A personal identification number was then assigned to each seed after receiving written informed consent from the participant. Next, we provided three coupons to each seed for referring three other people who use drugs to the study. Seeds received US$2 for a successful referral and were expected to refer three to six peers. New seeds would be selected when the recruitment tree had dried up. Finally, participants recruited for the study were invited to become seeds allowing them to recruit other people who use drugs from their networks.

**Fig. 1:**
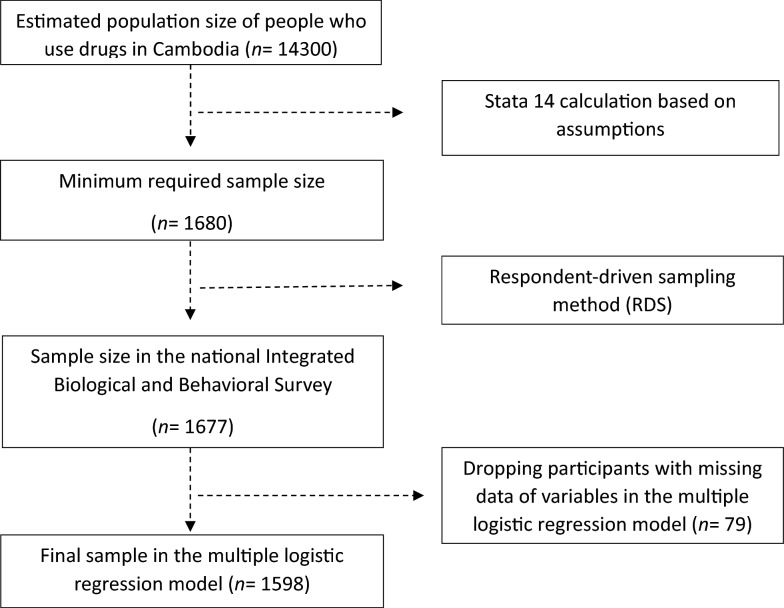
Flow chart of sample selection

Figure 1 shows the flow chart of the sample selection. The survey included 1677 participants. We finally included 1598 participants in the multiple logistic regression analysis after dropping 79 observations with missing data on the model variables.

### Data collection training

The interviews were conducted by formally trained counselors from HIV voluntary counseling and testing centers in the respective province. Data collection teams received 3 day training on data collection procedures, informed consent process, data collection tools, interview techniques, participants’ privacy and confidentiality protection, and data quality assurance.

### Variables and measurements

A structured questionnaire was developed based on standardized and validated tools adapted from previous studies on mental health among HIV key populations, including people who use drugs [21, 40–42]. We also conducted a questionnaire validation workshop participated by representatives from communities, NGOs, development partners, and national programs working on HIV and harm reduction in Cambodia. The questionnaire was piloted with 20 people who use drugs residing in Phnom Penh. The questionnaire collected information on sociodemographic characteristics, drug use behaviors, sexual behaviors, HIV and other sexually transmitted infections (STIs), other substance use, adverse childhood experiences, and psychological distress.

Sociodemographic characteristics included age (18–24, 25–34, 35 and older), sex (male, female), type of community (urban, rural), years of formal schooling attained (0–6, 7–9, 10 + years), average monthly income earned in the past 6 months (< 100, 100–199, 200 + USD), primary occupation (entertainment workers, office workers, laborer/farmer, unemployed, other), and living arrangement (living with family/relatives, living with friends, living in own dwelling, living on the streets, other).

The information on drug use included types of illicit drugs most commonly used and use frequency in the past 3 months. We also collected information regarding other substance use (i.e., alcohol drinking and binge drinking, cigarette smoking) and exposure to community-based HIV, harm reduction, and other related services in the past 6 months.

To measure HIV risks, we asked participants about their sexual behaviors in the past 3 months. The information included the number of sexual partners and condom use with commercial (defined as partners with whom the participant had sex in exchange for money or gifts) and non-commercial partners. We also collected information on HIV testing history, STI symptoms, and care-seeking behaviors for the symptoms in the past 3 months.

We adapted five ACE questions from the brief screening version of the Childhood Traumatic Questionnaire [43]. The items collected information on physical abuse, emotional abuse, sexual abuse, physical neglect, and emotional neglect. All five questions were close-ended, where respondents chose between zero (No) and one (Yes) to describe their ACEs.

We assessed psychological distress, the study’s outcome of interest, using the General Health Questionnaire (GHQ-12) [44]. A four-point Likert-type scale, which varied from “0 = less than usual” to “3 = much more than usual,” was applied to each question [44, 45]. A dichotomous variable of “0 = low psychological distress” and “1 = high psychological distress” was developed based on the GHQ-12 guide [45]. The four-point Likert-like scale was re-coded using a “0–0-1–1” method to eliminate bias [46]. The mean of the newly derived score’s sum was used as a cut-off to define participants with high (GHQ-12 > 2) and low (GHQ-12 ≤ 2) psychological distress [44]. Cronbach's alpha of the scale in this study was 0.88, confirming good reliability [47].

### Statistical analyses

Stata (StataCorp LP, version 14.2) was used for data analyses in this study. We compared sociodemographic characteristics, substance use, sexual behaviors, and ACEs of participants with low psychological distress (GHQ-12 ≤ 2) to those of participants with high psychological distress (GHQ-12 > 2). We used the Chi-square test (or Fisher's exact test when the expected cell count was smaller than five) for categorical variables and Student's *t*-test (or Mann–Whitney test when a variable was not normally distributed) for continuous variables. A multiple logistic regression model was constructed to identify factors associated with psychological distress. We included age and sex regardless of their statistical significance level and other variables associated with psychological distress in bivariate analyses at a level of *p*-value < 0.05 in the multiple logistic regression model. We obtained crude odds ratios (OR) and adjusted odds ratios (AOR) of the associations and presented with 95% confidence intervals (CIs) and *p*-values.

### Ethical considerations

This study received ethical approval from the National Ethics Committee for Health Research (NECHR) of the Ministry of Health in Cambodia (No. 420 NECHR). Participation in the study was voluntary, and all participants provided written informed consent. We protected participants’ privacy and confidentiality by collecting data in a private room and removing personal identifiers from research documents.

## Results

This study included 1598 people who use drugs, after excluding 79 participants with missing data. As shown in Table S1 (Additional file 1), no significant difference was found in the comparison of sociodemographic characteristics and psychological distress of the included and excluded samples. The participants included in the analyses had an average age of 28.6 years (SD = 7.8), average years of formal schooling completed of 6.0 years (SD = 3.9), and a median monthly income in the past 6 months of US$100.0 (interquartile range [IQR]: 60–150). People who inject drugs constituted 19% of the study sample. Around one-third of the participants resided in Phnom Penh, while 16% lived in Banteay Meanchey and 11% in Battambang province, which border Thailand. Of the total sample, 42% had high psychological distress – 50% in women and 37% in men.

The participants’ sociodemographic characteristics, substance use, sexual behaviors, and adverse childhood experiences are respectively presented in Tables S2, S3, S4, and S5 of the Additional file 1. In brief, approximately 63% of the participants were male, and 89% lived in an urban setting. More than half (53%) had primary or no formal education, and 66% were 25 years of age or older. The majority (95%) were in the Khmer ethnic group, and 46% were never married. The proportion of participants having been to a prison and a drug rehabilitation center in the past 12 months was 11 and 16%, respectively. More than half (55%) reported having been slapped, kicked, or received physical punishment from a family member or a guardian when they were growing up. About half (51%) reported having been insulted by family members or guardians, and 22% reported having been sexually harassed or abused when they were growing up.

### Factors associated with psychological distress

Results of bivariate and multiple logistic regression analyses are presented in Table 1. After adjustment for other covariates in the model, the odds of having high psychological distress was significantly higher among participants who were 25–34 years old (AOR 1.30, 95% CI 1.01–1.70) and 35 years and older (AOR 1.68, 95% CI 1.19–2.35), had been to a drug rehabilitation center (AOR 2.06, 95% CI 1.48–2.86), had been insulted by family members (AOR 2.09, 95% CI 1.62–2.70), and had been sexually harassed or abused by someone (AOR 1.80, 95% CI 1.38–2.36). The odds of having high psychological distress was significantly lower among participants who were male (AOR 0.53, 95% CI 0.41–0.69), lived in own dwelling (AOR 0.56, 95% CI 0.41–0.77), reported injecting as the mode of the first drug use (AOR 0.56, 95% CI 0.34–0.91), and had someone taking care of them when they got sick when they were growing up (AOR 0.68, 95% CI 0.47–0.99).

**Table 1:** Factors associated with psychological distress among people who use drugs in bivariate and multivariable logistic regression analyses (*n* = 1598)

Variables in the model	GHQ-12 ≤ 2 (*n* = 927)	GHQ-12 > 2 (*n* = 671)	Bivariate logistic regression	Multiple logistic regression
	*n* (%)	*n* (%)	OR (95% CI)	AOR (95% CI)
Sex				
Female	298 (32.1)	298 (44.4)	Reference	Reference
Male	629 (67.9)	373 (55.6)	0.59 (0.48–0.73)***	0.54 (0.41–0.69)***
Age group				
< 25	351 (37.8)	200 (29.8)	Reference	Reference
25–34	380 (41.0)	300 (44.7)	1.38 (1.10–1.74)**	1.31 (1.00–1.72)*
≥ 35	196 (21.2)	171 (25.5)	1.53 (1.17–2.00)**	1.65 (1.18–2.32)**
Current marital status				
Married	349 (37.6)	251 (37.4)	Reference	Reference
Never married	448 (48.3)	287 (42.8)	1.42 (1.06–1.90)*	1.01 (0.76–1.33)
Divorced/separated	130 (14.1)	133 (19.8)	0.89 (0.71–1.11)	1.08 (0.78–1.49)
Level of formal education completed				
Primary education or none (0–6)	472 (50.9)	382 (56.9)	Reference	Reference
Lower secondary education (7–9)	265 (28.6)	182 (27.1)	0.84 (0.67–1.07)	1.06 (0.82–1.37)
Upper secondary or higher (≥ 10)	190 (20.5)	107 (15.9)	0.69 (0.53–0.91)**	0.90 (0.66–1.23)
Type of living arrangements				
With family/relatives	438 (47.2)	314 (46.8)	Reference	Reference
On the streets	87 (9.4)	72 (10.7)	1.15 (0.82–1.63)	0.70 (0.46–1.06)
In own dwelling	230 (24.8)	131 (19.5)	0.79 (0.61–1.03)	0.56 (0.41–0.76)***
With friends	94 (10.2)	72 (10.8)	1.07 (0.76–1.50)	0.88 (0.61–1.27)
Others	78 (8.4)	82 (12.2)	1.47 (1.04–2.06)***	1.11 (0.76–1.63)
Duration of drug use				
≤ 2 years	584 (63.0)	380 (56.6)	Reference	Reference
3–5 years	187 (20.2)	147 (21.9)	1.21 (0.94–1.55)	1.19 (0.90–1.57)
6–9 years	54 (5.8)	44 (6.6)	1.25 (0.82–1.90)	1.07 (0.67–1.71)
≥ 10 years	102 (11.0)	100 (14.9)	1.51 (1.11–2.04)***	1.11 (0.75–1.63)
Had been sent to a drug rehabilitation center				
No	821 (88.6)	521 (77.6)	Reference	Reference
Yes	106 (11.4)	150 (22.4)	2.23 (1.70–2.93)***	2.05 (1.47–2.85)***
Injection as mode of first drug use				
No	838 (90.4)	609 (90.8)	Reference	Reference
Yes	89 (9.6)	62 (9.2)	0.95 (0.68–1.35)	0.57 (0.35–0.92)*
Had been sent to prison in the past 12 months				
No	834 (90.0)	583 (86.9)	Reference	Reference
Yes	93 (10.0)	88 (13.1)	1.35 (0.99–1.84)	0.99 (0.69–1.43)
Had been slapped, kicked by parents/guardians				
No	477 (51.5)	250 (37.3)	Reference	Reference
Yes	450 (48.5)	421 (62.7)	1.78 (1.45–2.18)***	1.13 (0.88–1.46)
Had been insulted by family members				
No	535 (57.7)	245 (36.5)	Reference	Reference
Yes	392 (42.3)	426 (63.5)	2.37 (1.93–2.91)***	2.10 (1.63–2.71)***
Had been sexually harassed/abused by someone				
No	780 (84.1)	468 (69.7)	Reference	Reference
Yes	147 (15.8)	203 (30.3)	2.3 (1.81–2.93)***	1.80 (1.38–2.35)***
Had someone taking care of them when they got sick				
No	86 (9.3)	83 (12.4)	Reference	Reference
Yes	841 (90.7)	588 (87.6)	0.72 (0.53–0.99)*	0.69 (0.48–1.01)*
Had emotional support from family members				
No	124 (13.4)	111 (16.5)	Reference	Reference
Yes	803 (86.6)	560 (83.5)	0.78 (0.59–1.03)	1.19 (0.86–1.65)
Injected drugs in the past 12 months				
No	769 (83.0)	531 (79.1)	Reference	Reference
Yes	158 (17.0)	140 (20.9)	1.28 (0.99–1.65)	1.40 (0.95–2.06)
Current HIV status				
Negative	588 (63.4)	430 (64.1)	Reference	Reference
Positive	31 (3.3)	47 (7.0)	2.07 (1.29–3.32)**	1.63 (0.98–2.71)
Do not know	308 (33.3	194 (28.9)	0.86 (0.69–1.07)	1.10 (0.87–1.40)

*AOR* adjusted odds ratio, *CI* confidence interval, *GHQ* General Health Questionnaire, *OR* odds ratio

Psychological distress was measured using the General Health Questionnaire (GHQ-12), and a total score of GHQ-12 > 2 was used to define high psychological distress

*p < 0.05; **p < 0.01, ***p < 0.001

## Discussion

This study provides evidence of the prevalence and factors associated with psychological distress, measured by GHQ-12, among people who use drugs in a resource-constrained country. We found that the prevalence of psychological distress among people who use drugs in this study was 42%, which was similar to the finding in our previous study conducted in 2014 using the same measure of psychological distress among people who inject drugs in the capital city of Phnom Penh [21].

### Sociodemographic factors

Our findings suggested that male people who use drugs were less likely to have high psychological distress than their female counterparts. This finding is consistent with the results shown in previous studies [21, 25, 48]. In Taiwan, female people who use drugs were more likely to have suicidal thoughts than their male counterparts, resulting in more psychiatric illnesses than men [48]. Finding in our setting could be explained by the differences in education level, type of occupation, and level of stigma and discrimination between male and female participants. In our bivariate analyses, individuals with upper secondary education were twice as less distressed as those with primary or no education. Only 7% of female participants had upper secondary education compared to 25% of men. We also found that 43% of female participants in this study worked in the entertainment sector compared to only 4% of men. Being female entertainment workers could be an underlying factor because a previous study among female entertainment workers in Cambodia showed a high prevalence of gender-based violence at work and its positive association with psychological distress [49]. This result implies that interventions to address mental health among people who use drugs should be gender-sensitive and focused on subpopulations with lower education and in occupations that expose them to underlying risks.

People who use drugs aged 25 years and older were significantly more likely to have high psychological distress than their younger peers aged 18 to 24. This finding corroborates with findings in a previous study in Cambodia [25]. In this study, we found that participants aged 25 and older were more likely to have suicidal thoughts and to drink alcohol four times or more per week in the past 3 months than their younger counterparts (aged 18–24). The co-occurrence of substance use and mental illness among people who use drugs [11–14] and the association between suicidal thoughts and mental health [3, 21] has also been documented in previous studies. An institutionalized experience, such as being sent to a drug rehabilitation center, could be another factor in this setting. In this study, around one in five of participants in the age groups of 25–34 years and 35 years and older had been sent to rehabilitation centers compared with one in ten among participants aged 18–24 years. In bivariate analyses, individuals with rehabilitation experience were more than twice more likely to have psychological distress than those without the experience. This result suggests that multi-faceted (e.g., the trauma from being institutionalized, multiple substance abuse) interventions should target the elder subpopulation of people who use drugs.

We found that people who use drugs living in their own dwelling were less distressed than those living with family. Our descriptive data showed that around 40% of the participants living in own dwelling and having been exposed to physical abuse during childhood exhibited psychological distress compared to 54% of those living with families and having had similar ACEs. Therefore, ACEs could have been a driver of the choice of living arrangement. It should be noted that marriage and cohabitation might have mediated the association as close to two-thirds of the participants living in their own dwelling were married and cohabited. Breaking free from the past ACEs with family and forming their independent cohabitation with a spouse, people who use drugs living in their own dwelling were less psychologically distressed than those living with family. This finding highlights the need for interventions that enhance families' roles in supporting behavioral changes in people who use drugs living with their families [50].

The finding that people who use drugs who had been to a drug rehabilitation center had more psychological distress than those who had never been to the center confirmed findings in previous studies in Cambodia [21, 25]. This finding suggests that the drug rehabilitation centers' role remained counterproductive 5 years later. The low overall quality of life and the health of people who use drugs with a history of rehabilitation could be the underlying drivers of psychological distress. However, adverse experience in rehabilitation centers remained a valid explanation, as indicated in another study in 2012 [25]. A recent investigation involving people who use drugs from two rehabilitation centers and relevant stakeholders in Phnom Penh suggested that there was evidence of physical abuse, inadequately-equipped facilities, absence of medical assistance to tackle withdrawal symptoms, non-existent counseling services, and absence of proper medical supervision [51]. Similar accounts had also been documented in China [52], Taiwan [48], and Vietnam [53]. It is important to note that living with HIV while in the rehabilitation centers might have compounded the participants’ psychological distress. A previous study in China reported a lack of ART access in rehabilitation centers among people who inject drugs living with HIV [52].

Moreover, the existing evidence showed that HIV-positive status is directly linked with mental disorders via neuro-biological mechanisms [54]. HIV-positive status is also indirectly related to mental disorders through stigma and discrimination [55], particularly among people living with HIV without access to antiretroviral therapy (ART) and insufficient or late ART receipt [56]. This result appeals to the policymakers to review the current roles and operation of drug rehabilitation centers and calls for adopting an alternative community-based program, as suggested in previous studies [25, 57].

### Adverse Childhood Experiences (ACEs)

The participants who had been insulted by family members or guardians or sexually harassed or abused when growing up exhibited a higher level of psychological distress than those who had not. On the other hand, the participants who experienced care provided by someone when they got sick were less psychologically distressed than those who did not. These findings are consistent with studies in the United States [58] and Cambodia [21]. In their systematic review, De Venter et al. showed that people who use drugs who had experienced ACEs exhibited symptoms or diagnoses of depressive and anxiety disorders [59]. The association’s pathway likely ran from ACEs to entry to drug use, and later, to psychological distress. Another study showed that people aged 14 and above in California with a higher ACE score were about two to four times more likely to initiate drug use than people with a lower ACE score [60]. Another possible pathway could have been from ACEs to low self-esteem or self-regards, drug use, and, finally, psychological distress. A study in Japan showed that female juvenile offenders who reported ACEs tended to have low self-esteem [61]. Another study in Iran showed that participants who had low self-esteem had a tendency towards drug addiction [62]. In Cambodia's setting, no research has empirically explored the association between ACEs and entry to drug use among the adult population, suggesting that future studies on this topic are warranted. Nevertheless, since ACEs are risk factors of psychological distress among adult people who use drugs, it is essential that these ACEs are identified and prevented [63].

### Limitations of the study

Despite several strengths, the limitations of this study should be noted. First, causal inference from our multiple logistic regression analysis could not be made as we did not address endogeneity (e.g., omitted variable bias or reverse causality) of each independent variable. Thus, the results should be interpreted as the association between the dependent and independent variables. Second, since our measure of psychological distress was constructed based on self-reported responses to the GHQ-12 questionnaire but not a performance-based psychological measure, our association results could be biased due to social desirability and recall bias. Third, the study findings’ generalizability may be limited because the study targeted provinces with a heavy HIV and drug use burden to obtain a large sample. Furthermore, the participants likely self-selected into the study as they could have been motivated by the incentive (token) provided through the RDS method.

## Conclusions

This study documents a high prevalence of and risk factors associated with psychological distress among people who use drugs in Cambodia. Risk factors significantly associated with psychological distress among people who use drugs in this study included sex, age, history of drug rehabilitation, and ACEs. Women showed higher psychological distress than men, while people aged 25 years and older also exhibited higher psychological distress than people aged 18–24. Our results on the counterproductive role of drug rehabilitation and the negative effect of ACEs on the psychological distress of people who use drugs also corroborated with findings in earlier studies. Therefore, intervention programs that attempt to address mental health among people who use drugs should be gender- and age-sensitive. The programs should be tailored to more vulnerable and marginalized subpopulations and individuals with a history of ACEs and drug rehabilitation. Importantly, integrating mental health services into HIV and harm-reduction programs for people who use drugs across all health care system levels is a promising alternative to tackle mental health problems among this vulnerable population. Community-based rehabilitation or treatment programs could be an alternative to rehabilitation centers, given the centers’ counterproductive roles.

## Supplementary information

**Additional file 1:** **Table S1. **Comparison of proportion of socio-demographics and psychological distress of the final sample and excluded sample. **Table S2.** Socio-demographic characteristics of people who use drugs with a high and low level of psychological distress. **Table S3**. Characteristics of substance use among people who use drugs with a high and low level of psychological distress. **Table S4.** Sexual behaviors among people who use drugs with a high and low level of psychological distress. **Table S5.** Gender-based violence and stigma exposure among people who use drugs with a high and low level of psychological distress.

## Data Availability

The data used for this study are owned by the National Center for HIV/AIDS, Dermatology and STD. They cannot be made available in the manuscript, the additional files, or a public repository. However, they can be accessed upon request from the Principal Investigator Dr. Siyan Yi (ephsyi@nus.edu.sg).
