# Supplementary material for: Prevalence and social determinants of psychological distress among people who use drugs in Cambodia
Source: Int J Ment Health Syst. 2020 Nov 4;14:77. doi: 10.1186/s13033-020-00411-5 (PMC7640420; doi:10.1186/s13033-020-00411-5)
Supplement: Supplementary file 1 — Additional file 1: Table S1. Comparison of proportion of socio-demographics and psychological distress of the final sample and excluded sample. Table S2. Socio-demographic characteristics of people who use drugs with a high and low level of psychological distress. Table S3. Characteristics of substance use among people who use drugs with a high and low level of psychological distress. Table S4. Sexual behaviors among people who use drugs with a high and low level of psychological distress. Table S5. Gender-based violence and stigma exposure among people who use drugs with a high and low level of psychological distress. [file 13033_2020_411_MOESM1_ESM.docx]

**Table S1 Comparison of proportion of socio-demographics and psychological distress of the final sample and excluded sample**

| **Characteristics** | | | **Final sample** | **Excluded sample^*^** |  |
| --- | --- | --- | --- | --- | --- |
|  | |  | **(*n*= 1598)** | **(*n*= 79)** |  |
|  | |  | ***n* (%)** | ***n* (%)** | ***P*-value***^†^* |
| Psychological distress (GHQ-12>2) | | | 671 (41.9) | 32 (40.5) | 0.79 |
| Living in an urban area | | | 1415 (88.5) | 70 (88.6) | 0.99 |
| Male | | | 1002 (62.7) | 51 (64.6) | 0.74 |
| Age groups | | |  |  |  |
|  | 18- 24 | | 551 (34.5) | 24 (31.6) | 0.60 |
|  | 25-34 | | 680 (42.5) | 36 (47.4) | 0.41 |
|  | ≥35 | | 367 (23.0) | 16 (21.0) | 0.70 |
| Khmer ethnic group | | | 1506 (94.6) | 72 (92.3) | 0.37 |
| Current marital status | | |  |  |  |
|  | Never married | | 735 (46.0) | 34 (51.5) | 0.38 |
|  | Married | | 600 (37.5) | 24 (36.4) | 0.85 |
|  | Widowed/divorced/separated | | 263 (16.5) | 8 (12.1) | 0.35 |
| Level of formal schooling completed | | |  |  |  |
|  | Primary education (0–6 years) | | 854 (53.4) | 45 (57.0) | 0.54 |
|  | Lower secondary (7–9 years) | | 447 (28) | 15 (19.0) | 0.08 |
|  | High school or higher (≥10 years) | | 297 (18.6) | 19 (24.0) | 0.22 |
| Living arrangement | | |  |  |  |
|  | With family or relatives | | 752 (47.1) | 29 (38.7) | 0.15 |
|  | In the streets | | 159 (9.9) | 12 (16.0) | 0.09 |
|  | In own dwelling | | 361 (22.6) | 21 (28.0) | 0.27 |
|  | With friends | | 166 (10.4) | 6 (8.0) | 0.51 |
|  | Other | | 160 (10.0) | 7 (9.3) | 0.85 |
| Main occupation | | |  |  |  |
|  | Unemployed | | 183 (11.5) | 14 (17.7) | 0.09 |
|  | Entertainment worker | | 291 (18.3) | 15 (19.0) | 0.86 |
|  | Office worker | | 76 (4.7) | 3 (3.8) | 0.69 |
|  | Laborer/farmer | | 573 (35.8) | 24 (30.4) | 0.32 |
|  | Other | | 475 (29.7) | 23 (29.1) | 0.91 |
| Average monthly income in the past 6 months (US$) | | | | | |
|  | <100 | | 563 (35.2) | 42 (53.2) | 0.001 |
|  | 100-199 | | 754 (47.2) | 26 (32.9) | 0.01 |
|  | ≥200 | | 282 (17.6) | 11 (13.9) | 0.40 |

*GHQ, General Health Questionnaire.*

*^*^Participants excluded from the analyses due to missing data of variables included in the analyses.*

*^†^Chi-square test was used to compare difference in proportion of socio-demographic between two samples.*

**Table S2 Socio-demographic characteristics of people who use drugs with a high and low level of psychological distress**

| **Sociodemographic characteristics** | | **Total**  **(*n*= 1598)** | **Level of psychological distress^*^** | | |
| --- | --- | --- | --- | --- | --- |
|  |  |  | **GHQ-12> 2 (*n*= 671)** | **GHQ-12≤ 2**  **(*n*= 927)** | |
|  |  | ***n* (%)** | ***n* (%)** | ***n* (%)** | ***P*-value^†^** |
| Living in an urban area | | 1415 (88.5) | 593 (88.4) | 822 (88.7) | 0.85 |
| Male | | 1002 (62.7) | 373 (55.6) | 629 (67.8) | <0.001 |
| Age groups | | | | | 0.003 |
|  | 18- 24 | 551 (34.5) | 200 (29.8) | 351 (37.9) |  |
|  | 25-34 | 680 (42.5) | 300 (44.7) | 380 (41.0) |  |
|  | ≥35 | 367 (23.0) | 171 (25.5) | 196 (21.1) |  |
| Ethnicity | | | | | 0.016 |
|  | Khmer | 1506 (94.7) | 643 (96.3) | 863 (93.5) |  |
|  | Vietnam | 85 (5.3) | 25 (3.7) | 60 (6.5) |  |
| Current marital status | | | | | 0.005 |
|  | Never married | 735 (46.0) | 287 (42.8) | 448 (48.3) |  |
|  | Married | 600 (37.6) | 251 (37.4) | 349 (37.6) |  |
|  | Widowed/divorced/separated | 263 (16.4) | 133 (19.8) | 130 (14.1) |  |
| Level of formal schooling completed | | | | | 0.026 |
|  | Primary education (0–6 years) | 854 (53.4) | 382 (56.9) | 472 (50.9) |  |
|  | Lower secondary (7–9 years) | 447 (28.0) | 182 (27.1) | 265 (28.6) |  |
|  | High school or higher (≥10 years) | 297 (18.6) | 107 (16.0) | 190 (20.5) |  |
| Living arrangement | | | | | 0.023 |
|  | With family or relatives | 752 (47.1) | 314 (46.8) | 438 (47.2) |  |
|  | In the streets | 159 (9.9) | 72 (10.7) | 87 (9.4) |  |
|  | In own dwelling | 361 (22.6) | 131 (19.5) | 230 (24.8) |  |
|  | With friends | 166 (10.4) | 72 (10.7) | 94 (10.1) |  |
|  | Other | 160 (10.0) | 82 (12.2) | 78 (8.4) |  |
| Main occupation | | | | | 0.037 |
|  | Unemployed | 183 (11.4) | 70 (10.4) | 113 (12.2) |  |
|  | Entertainment worker | 291 (18.2) | 145 (21.6) | 146 (15.7) |  |
|  | Office worker | 76 (4.8) | 29 (4.3) | 47 (5.1) |  |
|  | Laborer/farmer | 573 (35.9) | 240 (35.8) | 333 (35.9) |  |
|  | Other | 475 (29.7) | 187 (27.9) | 288 (31.1) |  |
| Average monthly income in the past 6 months (US$) | | | | | 0.365 |
|  | <100 | 563 (35.2) | 247 (36.8) | 316 (34.1) |  |
|  | 100-199 | 754 (47.2) | 315 (46.9) | 439 (47.4) |  |
|  | ≥200 | 281 (17.6) | 109 (16.2) | 172 (18.5) |  |

*GHQ, General Health Questionnaire.*

*^*^Psychological distress was measured using the General Health Questionnaire (GHQ-12), and a total score of GHQ-12> 2 was used to define high psychological distress.*

**^†^***Chi-square (or Fisher’s exact test when a cell count was smaller than 5) was used.*

**Table S3 Characteristics of substance use among people who use drugs with a high and low level of psychological distress**

| **Characteristics of substance use** | | **Total**  **(*n*= 1598)** | **Level of psychological distress^*^** | | |
| --- | --- | --- | --- | --- | --- |
|  |  |  | **GHQ-12> 2 (*n*= 671)** | **GHQ-12≤ 2**  **(*n*= 927)** | |
|  |  | ***n* (%)** | ***n* (%)** | ***n* (%)** | ***P*-value^†^** |
| Median months using drugs (IQR) | | 18 (7-48) | 24 (7-60) | 14 (6-48) | 0.005 |
| Mode of first drug use – injecting | | 151 (9.4) | 62 (9.2) | 89 (9.6) | 0.37 |
| First-time drug use was introduced to drugs by: | | | | | 0.57 |
|  | Myself | 245 (15.3) | 105 (15.6) | 140 (15.1) |  |
|  | Friends/relatives | 1277 (79.9) | 530 (79.0) | 747 (80.6) |  |
|  | Other (sexual partner, stranger) | 76 (4.8) | 36 (5.4) | 40 (4.3) |  |
| Used any drugs in the past 3 months | | 1262 (79.7) | 569 (85.4) | 693 (75.6) | <0.001 |
| Type of drugs most commonly used in the past 3 months | | | | |  |
|  | Heroin | 176 (11.0) | 83 (12.4) | 93 (10.0) | 0.14 |
|  | Yama/ice (methamphetamine) | 1165 (72.9) | 523 (77.9) | 642 (69.3) | <0.001 |
|  | Ecstasy | 64 (4.0) | 38 (5.7) | 26 (2.8) | 0.004 |
|  | Inhalants | 48 (3.0) | 31 (4.6) | 17 (1.8) | 0.001 |
| Injected drugs in the past 3 months | | 116 (7.3) | 56 (8.3) | 60 (6.5) | 0.15 |
| Always used new syringes/needles | | 113 (7.1) | 51 (7.6) | 62 (6.7) | 0.48 |
| Used needles/syringes used by someone else in the past 3 months | | 41 (2.6) | 25 (3.7) | 16 (1.7) | 0.013 |
| Had been to a prison in the past 12 months | | 181 (11.3) | 88 (13.1) | 93 (10.0) | 0.055 |
| Had been sent to a drug rehabilitation center in the past 12 months | | 256 (16.0) | 150 (22.3) | 106 (11.4) | <0.001 |
| Daily alcohol drinking habit (*n*= 1320) | |  |  |  | 0.017 |
|  | 1-2 cans per day | 310 (24.6) | 110 (20.6) | 200 (27.7) |  |
|  | 3-4 cans per day | 342 (27.2) | 145 (27.1) | 197 (27.2) |  |
|  | 5-6 cans per day | 315 (25.0) | 150 (28.0) | 165 (22.8) |  |
|  | At least 7 cans per day | 291 (23.1) | 130 (24.3) | 161 (22.3) |  |
| Smoked at least 100 cigarettes in lifetime | | 803 (50.3) | 344 (51.3) | 459 (49.6) | 0.48 |

*GHQ, General Health Questionnaire; IQR, interquartile range.*

*^*^Psychological distress was measured using the General Health Questionnaire (GHQ-12), and a total score of GHQ-12> 2 was used to define high psychological distress.*

**^†^***Chi-square (or Fisher’s exact test when a cell count was smaller than 5) was used for categorical variables and Mann-Whitney U test for continuous variables.*

**Table S4 Sexual behaviors among people who use drugs with a high and low level of psychological distress**

| **Sexual behaviors in the past 3 months** | **Total**  **(*n*= 1598)** | **Level of psychological distress** | | |
| --- | --- | --- | --- | --- |
|  |  | **GHQ-12> 2 (*n*= 671)** | **GHQ-12≤ 2^*^**  **(*n*= 927)** | |
|  | ***n* (%)** | ***n* (%)** | ***n* (%)** | ***P*-value^†^** |
| Had sexual intercourse | 1486 (93.0) | 642 (95.7) | 884 (91.0) | <0.001 |
| Median number of sex partners (IQR) | 1 (0-2) | 1 (1-3) | 1 (0-2) | 0.001 |
| Always used condom with any partner | 278 (25.2) | 125 (25.4) | 153 (25.0) | 0.55 |
| Had sex when a partner was intoxicated | 436 (39.7) | 242 (49.5) | 194 (31.9) | <0.001 |
| Had sex with partners not in exchange for money or gift | 601 (54.4) | 293 (59.7) | 308 (50.2) | 0.002 |
| Always used condom with partners not in exchange for money or gift | 125 (20.6) | 58 (19.6) | 67 (21.5) | 0.13 |
| Had sex in exchange for money or gifts | 397 (35.8) | 203 (41.2) | 194 (31.5) | 0.001 |
| Always used condom with partners in exchange for money or gift | 155 (39.2) | 70 (34.6) | 85 (44.0) | 0.005 |

*GHQ, General Health Questionnaire; IQR, interquartile range.*

*^*^Psychological distress was measured using the General Health Questionnaire (GHQ-12), and a total score of GHQ-12> 2 was used to define high psychological distress.*

**^†^***Chi-square (or Fisher’s exact test when a cell count was smaller than 5) was used for categorical variables and Mann-Whitney U test for continuous variables.*

**Table S5 Gender-based violence and stigma exposure among people who use drugs with a high and low level of psychological distress**

| **Violence and stigma exposure or adverse childhood experience** | **Total**  **(*n*= 1598)** | **Level of psychological distress^*^** | | |
| --- | --- | --- | --- | --- |
|  |  | **GHQ-12> 2 (*n*= 671)** | **GHQ-12≤ 2**  **(*n*= 927)** | |
|  | ***n* (%)** | ***n* (%)** | ***n* (%)** | ***P*-value^†^** |
| Had been slapped, kicked or received physical punishment from parents | 871 (54.5) | 421 (62.7) | 450 (48.5) | <0.001 |
| Had been insulted by family members | 818 (51.2) | 426 (63.5) | 392 (42.3) | <0.001 |
| Had been sexually harassed by someone (e.g. touching, watching pornography or sexual abuse) | 350 (21.9) | 203 (30.2) | 147 (15.8) | <0.001 |
| Had been taken care of by someone (e.g. providing guardian or accompanying to medical center) | 1429 (89.4) | 588 (87.6) | 841 (90.7) | 0.047 |
| Had received emotional support and care from family member | 1363 (85.3) | 560 (83.5) | 803 (86.6) | 0.078 |

*GHQ, General Health Questionnaire.*

*^*^Psychological distress was measured using the General Health Questionnaire (GHQ-12), and a total score of GHQ-12> 2 was used to define high psychological distress.*

**^†^***Chi-square was used.*
